# Latent profiles of humanistic care competence among psychiatric nurses: a cross-sectional study

**DOI:** 10.3389/fpsyg.2026.1861314

**Published:** 2026-07-03

**Authors:** Li Dou, Hui Wang, Jie Dou, Quangang Zhang, Qi Zheng, Nana Liu, Peng Zhang

**Affiliations:** 1 School of Life Sciences, Xuzhou Medical University, Xuzhou, Jiangsu, China; 2 Huaibei People's Hospital (New Campus), Huaibei, Anhui, China; 3 School of Nursing, Bengbu Medical University, Bengbu, Anhui, China; 4 Xuzhou Oriental Hospital, Xuzhou Medical University, Xuzhou, Jiangsu, China

**Keywords:** empathy, humanistic care competence, latent profile analysis, nursing practice environment, psychiatric nurses

## Abstract

**Objective:**

Humanistic care competence is critical to ensuring the quality of care for psychiatric patients, yet it shows substantial individual heterogeneity. This study aimed to identify latent profiles of humanistic care competence among psychiatric nurses and explore its influencing factors to provide evidence for the development of targeted intervention strategies.

**Methods:**

A cross-sectional study was conducted among 288 psychiatric nurses recruited through convenience sampling. Data on demographic characteristics, humanistic care competence, empathy, work environment, and social support were collected using standardized questionnaires. Humanistic care competence was operationalized based on the Knowing, Courage, and Patience dimensions of the Caring Ability Inventory (CAI). Latent profile analysis was conducted using these three dimensions. Univariate analysis and binary logistic regression were subsequently performed to identify factors associated with latent class membership.

**Results:**

Two latent profiles were identified: a High humanistic care competence–Low Courage group (83.0%) and a Low humanistic care competence–High Courage group (17.0%). The High humanistic care competence–Low Courage group was characterized by higher Knowing and Patience and lower Courage, whereas the opposite pattern was observed in the Low humanistic care competence–High Courage group. Using the latter as the reference group, binary logistic regression showed that older age, female gender, higher empathy, a more favorable work environment, higher social support, and fewer night shifts significantly predicted membership in the High humanistic care competence–Low Courage group (all *p* < 0.05).

**Conclusion:**

Psychiatric nurses’ humanistic care competence can be categorized into two distinct latent profiles characterized by contrasting patterns of Knowing, Courage, and Patience. Older age, female gender, higher empathy, a supportive work environment, greater social support, and lower night shift frequency were associated with the higher competence profile.

## 1 Introduction

The World Health Organization reported that more than one billion people worldwide are living with mental health conditions, highlighting a substantial and growing global burden on mental health systems (World Health Organization, 2025). This escalating demand has increased pressure on mental health care delivery, particularly on psychiatric nursing professionals, whose roles have progressively expanded from traditional task-oriented functions to more complex responsibilities, including comprehensive needs assessment, individualized intervention, and continuous psychosocial support.

Nursing is fundamentally grounded in humanistic care, which integrates humanistic values into clinical practice to support patients’ psychological, emotional, and behavioral well-being (Watson, 2009). Centered on a therapeutic nurse–patient relationship, humanistic care aims to enhance patients’ capacity to cope with illness-related stress and promote recovery (Watson, 1985). Empirical evidence has consistently shown that patients express strong expectations for humanistic care, and its high-quality delivery is associated with improved patient satisfaction and quality of life (Liu et al., 2023). In psychiatric settings, these needs are particularly pronounced, as individuals with mental disorders often experience impairments in cognition, emotion, and social functioning, leading to heightened reliance on understanding, respect, and emotional support from healthcare providers (Lewandowski et al., 2024). Accordingly, strengthening psychiatric nurses’ humanistic care competence is essential to improve care quality and support patient rehabilitation.

Empathy has been widely recognized as a core psychological attribute underpinning humanistic care competence among nurses. It reflects the capacity to understand and share others’ emotional states and is shaped by the interaction of individual traits and environmental influences (Otsuka et al., 2024; Moreno-Poyato et al., 2021). International studies have identified empathy as a critical intrinsic determinant of humanistic care competence (Kuşlu et al., 2026; Ozdemir and Kaplan, 2024). In addition, empirical findings suggest that humanistic care levels among nurses and patients remain at a moderate level, indicating substantial room for improvement (Jian et al., 2022). Research in nursing students further demonstrates that humanistic care competence is associated with professional motivation, educational exposure, and clinical environment, and is positively correlated with ethical sensitivity; moreover, empathy exhibits certain demographic differences in this population (Zhang et al., 2024). From the patient perspective, satisfaction with humanistic care is also influenced by demographic and institutional factors such as age and hospital level (Liu et al., 2023). Collectively, these findings suggest that humanistic care competence is shaped by multi-level determinants; however, existing evidence remains fragmented and lacks an integrated framework to explain how these factors interact to influence individual differences in competence (Lin et al., 2026).

Most existing studies have adopted a variable-centered approach, treating humanistic care competence as a homogeneous and continuously distributed construct (Bakaç et al., 2023). While this approach provides information on average relationships between variables, it fails to capture potential heterogeneity within nursing populations and may overlook distinct subgroups with different competence patterns and risk profiles. Such limitations restrict the development of targeted and personalized intervention strategies. To address this gap, person-centered approaches such as Latent Profile Analysis (LPA) have been increasingly recommended. LPA is a model-based clustering technique that identifies unobserved subgroups within a population based on response patterns across multiple observed indicators (Marshman et al., 2022). Compared with traditional clustering methods, LPA provides a probabilistic classification framework with stronger statistical rigor, enabling more robust identification of latent heterogeneity in complex behavioral constructs.

Guided by Bronfenbrenner’s Ecological Systems Theory, which posits that individual development and behavior result from dynamic interactions across multiple environmental systems (Bronfenbrenner, 1979; Sallis et al., 2015), this study selected empathy, the nursing practice environment, and perceived social support to operationalize the individual, organizational, and social ecological levels, respectively. At the individual level, empathy was assessed using the Interpersonal Reactivity Index (IRI-C). At the organizational level, the nursing practice environment was evaluated using the Practice Environment Scale (PES), which reflects critical structural and managerial characteristics of clinical settings, including teamwork, leadership support, staffing adequacy, and resource availability. In this study, the total PES score was used to represent the overall organizational practice environment. At the social level, perceived social support was measured using the Social Support Rating Scale (SSRS), representing the broader social resources available to nurses. Empathy, organizational practice environment, and social support were each measured using validated instruments corresponding to the respective ecological levels. Together, these variables provide an integrated multi-level perspective on factors influencing humanistic care competence.

Accordingly, this study aimed to: (1) identify latent profiles of humanistic care competence among psychiatric nurses using Latent Profile Analysis; and (2) examine the associations between individual-, organizational-, and social-level factors and latent profile membership within the framework of Ecological Systems Theory. By doing so, it sought to advance the understanding of heterogeneity in humanistic care competence and provide evidence for the development of targeted, profile-specific intervention strategies in psychiatric nursing practice.

## 2 Materials and methods

### 2.1 Study participants

**Figure 1:**
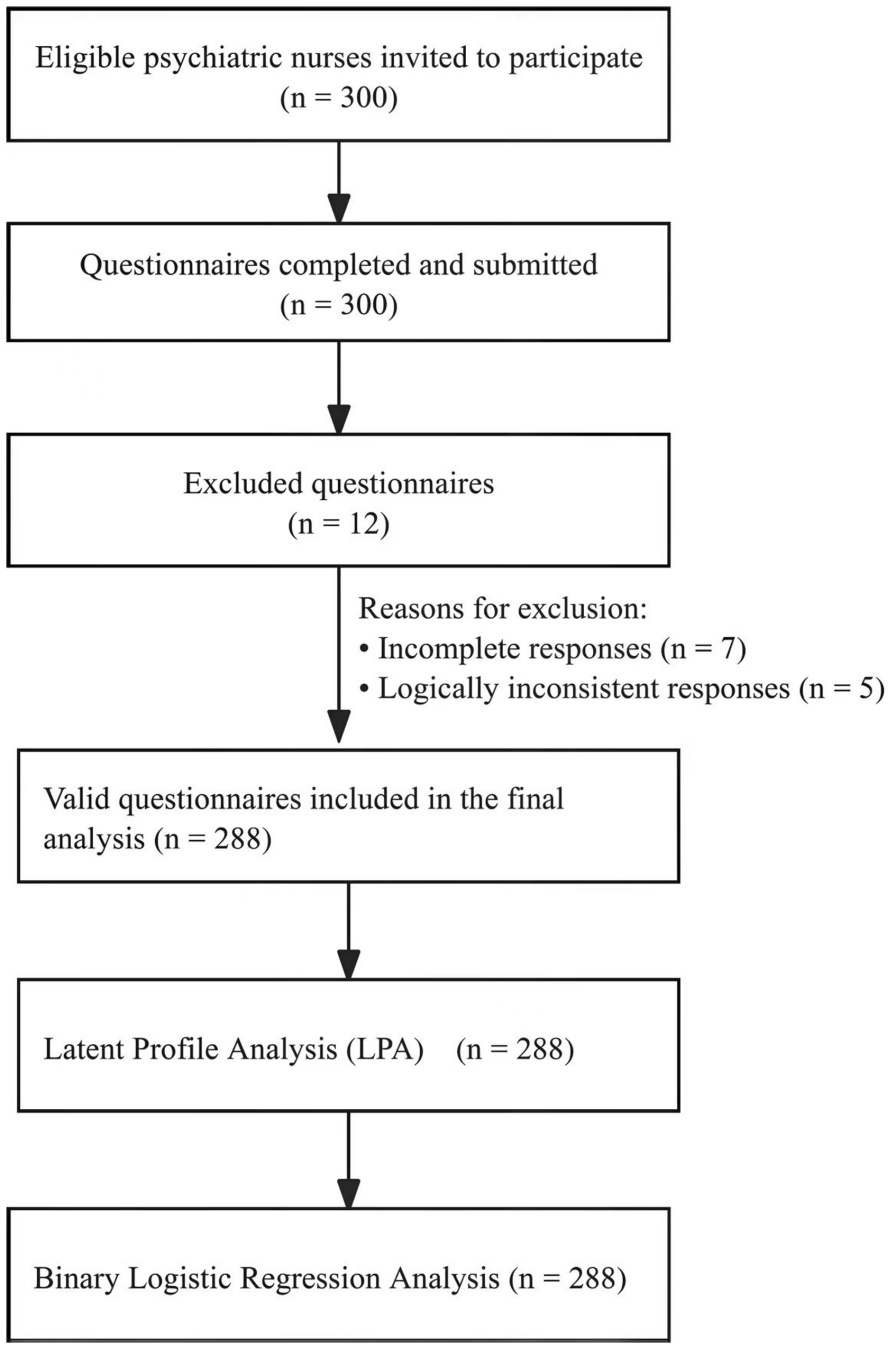
Flow diagram of participant recruitment, questionnaire screening, and inclusion in the final analyses.

This study employed a cross-sectional survey design. In January 2026, clinical psychiatric nurses were recruited using convenience sampling from two psychiatric hospitals in Xuzhou, Jiangsu Province, China. The participant recruitment and selection process is presented in Figure 1.

Inclusion criteria were as follows: (1) registered nurses with valid nursing practice certificates; (2) at least one year of continuous clinical work experience in psychiatric departments; (3) voluntary participation in the study; and (4) no severe physical diseases (e.g., heart failure, liver failure, or renal failure) or mental health disorders that could interfere with questionnaire completion.

Exclusion criteria were: (1) student interns, visiting nurses, or nurses on short-term rotation in psychiatric settings; (2) nurses on extended leave (e.g., maternity leave or sick leave exceeding one month) during data collection; and (3) participants who withdrew from the study or had more than 10% missing data on key questionnaire items.

#### 2.1.1 Sample size calculation

Sample size was considered based on methodological recommendations for latent profile analysis and regression analysis. For latent profile analysis (LPA), sample size requirements are not based on fixed numerical rules but depend on factors such as class separation, model complexity, and class proportions. Simulation studies have shown that class enumeration accuracy and classification stability in LPA are influenced by sample size, with larger samples generally yielding more stable solutions (Nylund et al., 2007; Tein et al., 2013). For the subsequent binary logistic regression analysis, a commonly used criterion of at least 10 events per predictor variable was considered (Peduzzi et al., 1996). Following univariate screening, 7 predictors were included in the final model. Overall, the final analytic sample (*N* = 288) is consistent with commonly reported sample sizes in exploratory latent profile studies and is considered sufficient to support the identification of a small number of latent profiles in the present study.

### 2.2 Research instruments

#### 2.2.1 General information questionnaire

This section was designed to collect demographic and work-related variables, including gender, age, marital status, educational background, professional title, and employment type.

#### 2.2.2 Caring Ability Inventory (CAI)

The Caring Ability Inventory (CAI), developed by Nkongho (2003), was used to assess humanistic care competence in interpersonal relationships. It consists of 37 items across three dimensions: Knowing (14 items), Courage (13 items), and Patience (10 items). The scale is rated on a 7-point Likert scale from 1 (strongly disagree) to 7 (strongly agree), with total scores ranging from 37 to 259, and higher scores indicating stronger caring ability. The Cronbach’s *α* coefficients for the Knowing, Courage, and Patience subscales were 0.732, 0.702, and 0.748, respectively. In the present study, the CAI demonstrated good internal consistency, with a Cronbach’s *α* coefficient of 0.870 for the total scale. A representative item is: “I am willing to spend time getting to know others.”

#### 2.2.3 Interpersonal Reactivity Index – Chinese version (IRI-C)

The Interpersonal Reactivity Index (IRI) was originally developed by Davis in 1980 (Davis, 1983) and comprises four dimensions: Fantasy, Personal Distress, Perspective-Taking, and Empathic Concern. The Chinese version of the IRI (IRI-C) (Zhang et al., 2010) retains the same four-factor structure and has been widely used among Chinese healthcare professionals, demonstrating satisfactory reliability and validity. The IRI-C consists of 22 items rated on a 5-point Likert scale, with higher scores indicating greater empathy. In the present study, the scale showed acceptable internal consistency (Cronbach’s *α* = 0.725). A representative item is: “I often have tender, concerned feelings for people less fortunate than me.”

#### 2.2.4 Practice Environment Scale (PES)

The Practice Environment Scale (PES) was developed by Lake (2002) to evaluate key characteristics of the hospital nursing practice environment (Chiang and Lin, 2009). The Chinese version of the PES was adopted in this study (Wang and Li, 2011). The scale includes five dimensions: nurse participation in hospital affairs, foundations for quality care, nurse manager ability and leadership, adequate staffing and resources, and collegial nurse–physician relations. It is rated on a 4-point Likert scale (1–4), with higher scores indicating a more favorable nursing practice environment. The scale demonstrated good internal consistency in the present study, with a Cronbach’s *α* coefficient of 0.912. A representative item is: “I have opportunities for professional development or clinical advancement.”

#### 2.2.5 Social Support Rating Scale (SSRS)

The Social Support Rating Scale (SSRS) was developed by Xiao (1994) in 1986 to assess social support levels. It includes 10 items across three dimensions: objective support, subjective support, and support utilization. The total score ranges from 12 to 66, with higher scores indicating higher perceived social support. The scale demonstrated acceptable internal consistency in the present study, with a Cronbach’s *α* coefficient of 0.720. A representative item is: “How many close friends do you have from whom you can seek support and help?”

### 2.3 Data collection and quality control

Following ethical approval, standardized online questionnaire links were distributed to eligible psychiatric nurses with the assistance of nursing department administrators. The first page of the questionnaire provided information regarding the study purpose, significance, data usage, and data confidentiality. Participants provided electronic informed consent before accessing the formal questionnaire. To ensure data quality, data validation checks were implemented, including mandatory responses for all items and a one-submission limit per IP address and device. A total of 300 questionnaires were distributed. After excluding 12 incomplete or logically inconsistent responses, 288 valid questionnaires were retained, yielding a response rate of 96%.

### 2.4 Statistical analysis

Latent Profile Analysis (LPA) was conducted using Mplus 8.4 software. Scores for the three dimensions of the Caring Ability Inventory (Knowing, Courage, and Patience) were used as observed indicators to establish the model. Models were sequentially fitted from 1 class up to a maximum of 5 classes. Multiple fit indices were used to determine the optimal class number: Akaike Information Criterion (AIC), Bayesian Information Criterion (BIC), and sample-size-adjusted BIC (aBIC). Lower values suggest better model fit. Entropy was used to evaluate classification precision; values closer to 1 indicate better separation, and values ≥ 0.8 suggest high classification accuracy. The Bootstrap Likelihood Ratio Test (BLRT) and Lo–Mendell–Rubin Adjusted Likelihood Ratio Test (LMR) were used to compare model fit between nested models. A significant *p*-value indicates that the k-class model fits significantly better than the (k–1)-class model.

All statistical analyses were performed using IBM SPSS 27.1 software. Normality assumptions for continuous variables were assessed using the Shapiro–Wilk or Kolmogorov–Smirnov test, as appropriate. Based on distributional characteristics, normally distributed variables were presented as mean ± standard deviation and analyzed using independent-samples t tests, whereas non-normally distributed variables were analyzed using the Kruskal–Wallis H test. Categorical variables were presented as frequencies and percentages, and between-group differences were examined using the chi-square test.

Prior to multivariable analysis, multicollinearity among independent variables was assessed using variance inflation factors (VIFs), all of which ranged from 1.029 to 1.241, indicating no evidence of multicollinearity. Variables with *p* < 0.05 in univariate analyses were entered into a binary logistic regression model to identify factors associated with latent profile membership.

Night shift frequency was treated as a categorical variable and dummy coded for regression analyses, with “0 night shifts per week” used as the reference category. Two dummy variables were created: “1–2 night shifts per week” (1 = yes, 0 = no) and “>2 night shifts per week” (1 = yes, 0 = no). Adjusted odds ratios (ORs) with 95% confidence intervals (CIs) were reported, and a two-sided *p* value < 0.05 was considered statistically significant.

### 2.5 Ethical approval

This study was approved by the Ethics Committee of Xuzhou Oriental Hospital, Xuzhou Medical University (Approval No. 202501024003). The purpose, procedures, potential risks, and benefits of the study were fully explained to all participants. All data were processed in strict accordance with ethical guidelines, and strict confidentiality and anonymity were guaranteed throughout the study.

## 3 Results

### 3.1 Selection of the optimal model and naming of categories

Latent profile models with one to five classes were initially estimated. The five-class solution failed to converge reliably and yielded a very small class size (*n* < 10), suggesting model instability and potential overfitting; therefore, it was excluded from further analyses. Consequently, four competing models (1- to 4-class solutions) were retained for comparison, and their fit indices are presented in Table 1.

**Table 1:** Fit indices for latent profile models of humanistic care competence among psychiatric nurses.

Model	AIC	BIC	aBIC	Entropy	LMR	BLRT	Categorical probability
1	6684.946	6706.924	6687.897	—	—	—	1
2	6399.314	6435.944	6404.233	0.929	0.006	<0.001	0.170/0.830
3	6330.994	6382.276	6337.88	0.945	0.147	<0.001	0.170/0.799/0.031
4	6202.976	6268.909	6211.829	0.926	0.140	<0.001	0.094/0.594/0.201/0.111

AIC, Akaike Information Criterion; BIC, Bayesian Information Criterion; aBIC, sample-size-adjusted Bayesian Information Criterion; BLRT, Bootstrap-based Likelihood Ratio Test; LMR, Lo–Mendell–Rubin corrected Likelihood Ratio Test.

Across models, AIC, BIC, and adjusted BIC values decreased progressively with increasing class numbers, indicating improved statistical fit. However, model selection was not based solely on information criteria, given their known tendency to continuously decrease with additional parameters. Therefore, multiple statistical and substantive indicators were jointly considered. Although the 3-class model showed the highest entropy (0.945), the Lo–Mendell–Rubin likelihood ratio test was not statistically significant (*p* = 0.147), indicating no significant improvement over the 2-class solution. In addition, one class in the 3-class model accounted for only 3.1% of the sample, limiting both its statistical stability and substantive interpretability.

In contrast, the 2-class model demonstrated a more optimal balance between model fit and interpretability. The Lo–Mendell–Rubin test indicated that the 2-class model fit significantly better than the 1-class solution (*p* = 0.006). It also showed high entropy (0.929), indicating good classification accuracy, and yielded two adequately sized and clearly distinguishable classes (83.0 and 17.0%), supporting its practical interpretability. Considering statistical fit, model parsimony, class separation, and substantive interpretability, the two-class solution was selected as the optimal model.

**Figure 2:**
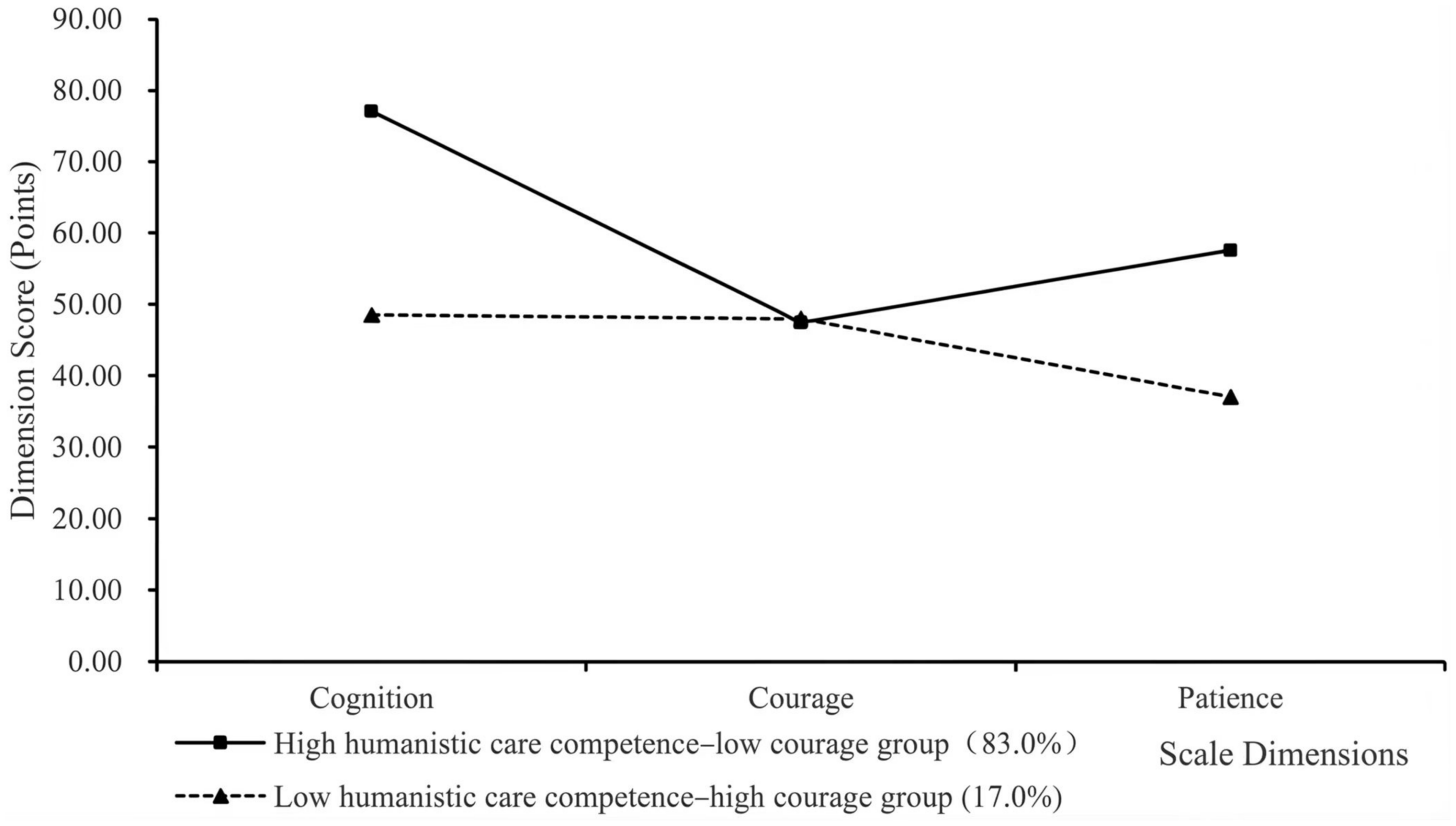
Latent profile patterns of humanistic care competence among psychiatric nurses.

As shown in Figure 2, the two latent classes exhibited distinct response patterns across the three dimensions of humanistic care competence. Based on the scoring profiles of the Knowing, Courage, and Patience dimensions of the Caring Ability Inventory (CAI), two latent profiles of humanistic care competence were identified and labeled consistently throughout the manuscript.

High humanistic care competence–low courage group (*n* = 239, 83.0%): This class was characterized by relatively higher scores on the Knowing (77.02) and Patience (57.64) dimensions and a relatively lower score on the Courage dimension (47.51). Because nurses in this class demonstrated stronger overall performance across the three dimensions despite comparatively lower Courage scores, it was labeled the “High humanistic care competence–low courage group.”

Low humanistic care competence–high courage group (*n* = 49, 17.0%): This class was characterized by relatively lower scores on the Knowing (48.55) and Patience (37.04) dimensions and a relatively higher score on the Courage dimension (48.04). Given its comparatively lower overall performance across the three dimensions despite relatively higher Courage scores, it was labeled the “Low humanistic care competence–high courage group.”

### 3.2 Comparative analysis of latent humanistic care profiles among psychiatric nurses

**Figure 3:**
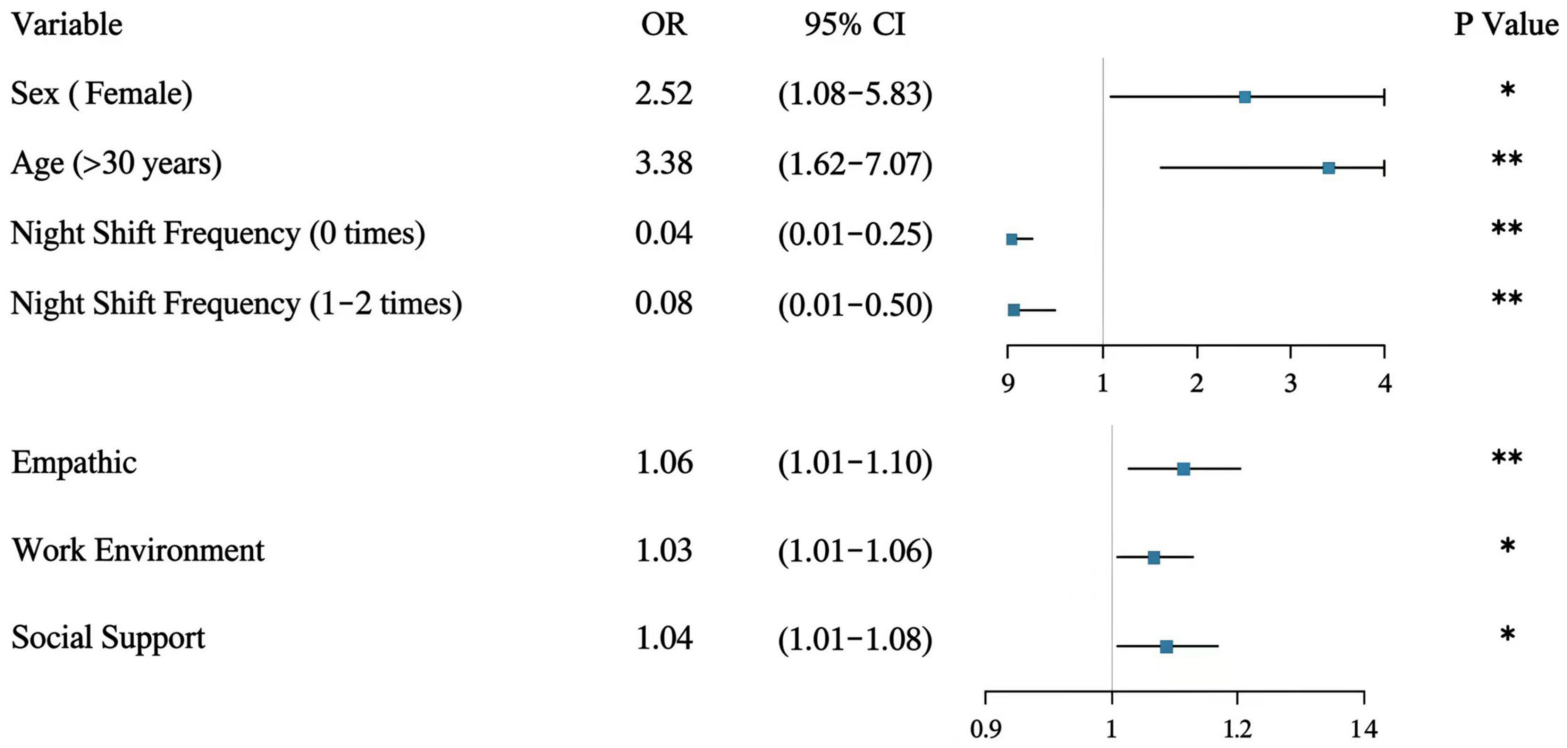
Adjusted odds ratios (ORs) and 95% confidence intervals (CIs) for factors associated with membership in the High humanistic care competence–low courage group (reference: Low humanistic care competence–high courage group).

The comparative analysis (Table 2) revealed significant differences between the High humanistic care competence–low courage group and the Low humanistic care competence–high courage group in gender, age, night shift frequency, empathic ability, work environment, and social support scores (all *p* < 0.05) (Figure 3).

**Table 2:** Comparison of demographic and study variables between two latent profiles of humanistic care competence among psychiatric nurses (*N* = 288).

Project	Group	Low humanistic care Competence–high courage group (*n* = 49)	High humanistic care Competence–low courage group (*n* = 239)	Statistics	*p*-value
Sex	Male	20 (40.8%)	38 (15.9%)	15.697a	<0.001
	Female	29 (59.2%)	201 (84.1%)		
Age	≤30	33 (67.3%)	98 (41%)	11.380a	0.001
	>30	16 (32.7%)	141 (59%)		
Educational level	Junior college	14 (28.6%)	62 (25.9%)	0.409a	0.815
	Undergraduate	28 (57.1%)	148 (61.9%)		
	Postgraduate	7 (14.3%)	29 (12.1%)		
Professional Title	Junior	17 (34.7%)	97 (40.6%)	0.830b	0.674
	Mid-Level	32 (65.3%)	139 (58.2%)		
	Senior	0 (0%)	3 (1.3%)		
Marital status	Unmarried	27 (55.1%)	112 (46.9%)	2.364b	0.287
	Married	20 (40.8%)	101 (42.3%)		
	other	2 (4.1%)	26 (10.9%)		
Night Shifts (shifts/week)	0	3 (6.1%)	39 (16.3%)	8.089a	0.018
	1–2	28 (57.1%)	152 (63.6%)		
	>2	18 (36.7%)	48 (20.1%)		
Employment type	Contract-based	37 (75.5%)	150 (62.8%)	2.910a	0.233
	Agency employment	8 (16.3%)	58 (24.3%)		
	Tenured position	4 (8.2%)	31 (13%)		
Nurse Empathy Scale		47.96 ± 9.568	52.54 ± 10.087	−2.920c	0.004
Practice Environment Scale		80.94 ± 16.536	86.36 ± 12.102	−2.671c	0.008
Social Support Rating Scale		35.49 ± 10.476	42.19 ± 9.587	−4.387c	<0.001

a: χ^2^ value (Chi-square value) b: Fisher’s exact test (Fisher’s exact probability); c: t value.

Binary logistic regression analysis was employed to investigate factors associated with latent class membership of humanistic care competence among psychiatric nurses, using the Low humanistic care competence–high courage group as the reference group. Variables that were statistically significant in the univariate analyses were entered into the regression model. Empathy, work environment, and social support scores were entered as continuous variables. Gender was coded as a binary variable (1 = female, 0 = male), with male serving as the reference category. Age was dichotomized as >30 years (1) and ≤30 years (0), with ≤30 years as the reference category. Night shift frequency was dummy coded using “0 night shifts per week” as the reference category. Two dummy variables were created: “1–2 night shifts per week” (1 = yes, 0 = otherwise) and “>2 night shifts per week” (1 = yes, 0 = otherwise) (Table 3).

**Table 3:** Factors associated with latent profile membership of humanistic care competence among psychiatric nurses: results of binary logistic regression analysis.

Variable	B	SE	Wald	OR	*p*
Sex (Female)	0.923	0.429	4.621	2.516 (1.085, 5.834)	0.032
Age (>30 years)	1.219	0.376	10.511	3.385 (1.620, 7.073)	0.001
Night Shift Frequency (0 times)	−3.255	0.955	11.613	0.039 (0.006, 0.251)	0.001
Night Shift Frequency (1–2 times)	−2.497	0.916	7.426	0.082 (0.014, 0.496)	0.006
Empathy	0.056	0.021	6.954	1.057 (1.014, 1.102)	0.008
Work Environment	0.034	0.014	5.863	1.034 (1.006, 1.063)	0.015
Social Support	0.043	0.019	5.071	1.044 (1.006, 1.084)	0.024
Constant	−4.465	1.673	7.127	0.012	0.008

The Low humanistic care competence–high courage group was used as the reference group in the binary logistic regression analysis. ORs represent the odds of membership in the High humanistic care competence–low courage group. OR > 1 indicates higher odds of belonging to the High humanistic care competence–low courage group, whereas OR < 1 indicates lower odds of belonging to this group.

## 4 Discussion

### 4.1 Heterogeneity in humanistic care competence among psychiatric nurses

High humanistic care competence–low courage group (83.0%): This was the dominant profile, accounting for 83.0% of the sample, indicating that this configuration is common among psychiatric nurses. Nurses in this group demonstrated higher scores on the Knowing and Patience dimensions, reflecting stronger abilities in understanding patients’ needs, recognizing emotions, and maintaining supportive humanistic care interactions. Although their Courage scores were slightly lower than those of the other group, the between-group difference was modest. Overall, this profile was associated with higher levels of humanistic care competence.

Low humanistic care competence–high courage group (17.0%): Nurses in this group showed lower scores on the Knowing and Patience dimensions, suggesting weaker abilities in understanding patients’ experiences and sustaining patient-centered humanistic care interactions. Although their Courage scores were relatively higher than those of the High humanistic care competence–low courage group, profile differentiation showed relatively greater differences in the Knowing and Patience dimensions. Lower Knowing scores further reflected reduced cognitive understanding within humanistic care competence. Overall, this profile was associated with lower levels of humanistic care competence.

The profile labels were determined based on the relative levels of CAI dimensions across latent classes, with “high” and “low” reflecting between-class comparisons rather than absolute values of any single dimension.

### 4.2 Factors associated with humanistic care competence among psychiatric nurses

Based on the identified latent profiles, with class differentiation showing relatively greater differences in the Knowing and Patience dimensions, we further examined factors associated with latent class membership.

#### 4.2.1 Age

Senior nurses were more likely to belong to the High humanistic care competence–low courage group (OR = 3.385, 95% CI: 1.620–7.073), consistent with previous studies (Yildirim et al., 2025)^.^ Age was associated with differences in clinical expertise, self-efficacy, professional commitment, and communication skills (Leal-Costa et al., 2020; Chen et al., 2023; Miao et al., 2025). These factors were associated with humanistic care competence. Structured mentorship programs may be considered in nursing management. Such programs may include experience sharing, role modeling, and reflective practice. These strategies may support the use of clinical experience as an organizational resource associated with humanistic care competence.

#### 4.2.2 Gender

Female nurses were more likely to be classified in the High humanistic care competence–low courage group (OR = 2.516, 95% CI: 1.085–5.834). This finding is interpreted in relation to social role expectations in nursing practice. Female nurses were more frequently assigned emotionally intensive care tasks. This pattern was associated with repeated clinical exposure and skill acquisition (Aca et al., 2025; Zhou et al., 2025). Higher empathy and emotional perception were also observed and may be associated with humanistic care behaviors (Chen et al., 2024). This result highlights the need to be mindful of the potential influence of gender role perceptions on task assignment in workforce management in order to promote the balanced development of competencies within nursing teams.

#### 4.2.3 Night shift frequency

Using the Low humanistic care competence–high courage group as reference, nurses with 0 night shifts (OR = 0.039, 95% CI: 0.006–0.251, *p* = 0.001) and 1–2 night shifts per week (OR = 0.082, 95% CI: 0.014–0.496, *p* = 0.006) were less likely to belong to the High humanistic care competence–low courage group. Night shift frequency was associated with differences in workload patterns and occupational strain. Circadian disruption, fatigue, and emotional exhaustion were observed conditions in night shift work. Frequent night shift work may be associated with cumulative occupational strain, which could contribute to differences in humanistic care competence (Brum et al., 2022). Night shift exposure was also associated with differences in perceived organizational support (Zhu and Mi, 2025). Adjustments in shift scheduling and support systems may be considered in clinical management.

#### 4.2.4 Empathy

Empathy was associated with humanistic care competence (OR = 1.057, 95% CI: 1.014–1.102). It reflects nurses’ capacity for perspective-taking and emotional resonance in nurse–patient interactions (Luo et al., 2024)^.^ Enhanced empathic processing may facilitate more accurate recognition of patients’ emotional states and support adaptive interpersonal responses, including attentive listening and emotional comfort. Through these mechanisms, empathy may strengthen the development of humanistic care competence (Decety et al., 2016). Accordingly, empathy may be considered an important attribute in nursing recruitment and training programs.

#### 4.2.5 Work environment

Work environment was positively linked to humanistic care competence. Adequate staffing, sufficient material resources, and supportive leadership structures may alleviate nurses’ emotional exhaustion (Teng et al., 2024). Beyond reducing occupational strain, a supportive practice environment may also foster higher levels of work engagement and altruistic motivation. In turn, these positive psychological states may encourage more consistent and sustained delivery of humanistic care behaviors. Overall, the work environment appears to function as an important organizational context shaping humanistic care competence (Wang et al., 2024). These findings suggest that optimizing organizational structure and strengthening leadership support may improve nursing care quality.

#### 4.2.6 Social support

Social support was positively related to humanistic care competence. Emotional and instrumental support from colleagues, supervisors, and family members may buffer occupational stress and reduce emotional exhaustion (Dziedzic et al., 2025). In addition, social support may contribute to enhanced psychological resilience and greater emotional stability (Liu et al., 2022). These psychosocial resources may, in turn, facilitate more empathic, respectful, and patient-centered care behaviors. Overall, stronger social support appears to be associated with more favorable humanistic care outcomes in psychiatric nursing practice. Therefore, strengthening team cohesion and promoting work–family balance may be beneficial in clinical settings.

## 5 Conclusion

This study identified significant heterogeneity in humanistic care competence among psychiatric nurses. Two distinct latent profiles with different competency patterns were identified using latent profile analysis. Multiple individual-, organizational-, and social-level factors were associated with profile membership. These findings highlight the importance of considering nurse heterogeneity when developing strategies to improve humanistic care competence and occupational well-being in psychiatric settings.

### 5.1 Study strengths and limitations

#### 5.1.1 Strengths

1. This study employed Latent Profile Analysis to identify, for the first time, distinct latent classes of humanistic care competence among psychiatric nurses. This approach moves beyond the traditional perspective of treating this ability as a homogeneous construct across the entire population, thereby facilitating a more precise identification of nurse subgroups with relatively insufficient caring capacity.
2. By integrating the Ecological Systems Theory, the research incorporated multi-level factors-including individual characteristics, organizational environment, and social support-into the analysis. This enabled the construction of a relatively comprehensive framework for understanding the influencing factors of humanistic care competence.
3. The findings offer valuable insights for psychiatric nursing management. They can provide an empirical basis for designing targeted, stratified, and categorized training and support strategies aimed at enhancing humanistic care.

#### 5.1.2 Limitations

1. The cross-sectional design and reliance on self-reported questionnaires preclude causal inferences, and the results may be influenced by social desirability bias and common method variance.
2. Limitations related to statistical methods and the sample: The class structure derived from latent profile analysis carries inherent uncertainty. Furthermore, the data were collected through online questionnaires distributed via hospital administrators, which may introduce potential selection bias, as nurses with higher engagement or willingness to participate may have been more likely to respond. In addition, the sample was drawn from two psychiatric hospitals in Xuzhou, Jiangsu Province, China. Given the regional concentration of the sample and the unique organizational and clinical characteristics of psychiatric specialty hospitals, the generalizability of the findings to other regions and healthcare settings may be limited.
3. Although the study is based on ecological systems theory, variable selection remained somewhat skewed toward the individual level. Macro-level factors such as organizational management and policy environment were not sufficiently examined.

#### 5.1.3 Conclusion

There is significant heterogeneity in humanistic care competence among psychiatric nurses, who can be divided into two latent profiles. Nursing managers should implement targeted and differentiated strategies to promote the humanistic care competence of psychiatric nurses according to the characteristics of different profiles. Future research could employ longitudinal tracking or multi-source data designs to overcome the limitations of cross-sectional and self-reported data. Secondly, expanding the study population to include nursing groups from different departments, hospital types, and cultural backgrounds would enhance the generalizability of the conclusions. Furthermore, integrating multi-level variables such as organizational management, team interaction, and policy environment could provide a stronger evidence base for nursing team development.

## Data Availability

The raw data supporting the conclusions of this article will be made available by the authors, without undue reservation.
